# Priorities for breast cancer risk assessment in UK women under age 50: a survey and discrete choice experiment

**DOI:** 10.1038/s41416-026-03546-3

**Published:** 2026-08-04

**Authors:** Rebecca A. Dennison, Maria Valasaki, Stuart Wright, Reuben Frost, Sacha J. Howell, Antonis C. Antoniou, Douglas F. Easton, Stephen Morris, Montserrat Garcia-Closas, Victoria G. Woof, David P. French, Juliet A. Usher-Smith

**Affiliations:** 1 Primary Care Unit, Department of Public Health and Primary Care, University of Cambridge, Cambridge, UK; 2 Manchester Centre for Health Economics, Division of Population Health, Health Services Research and Primary Care, School of Health Sciences, Faculty of Biology, Medicine and Health, University of Manchester, Manchester, UK; 3 Division of Genetics and Epidemiology, The Institute of Cancer Research, London, UK; 4 Division of Cancer Sciences, Faculty of Biology, Medicine and Health, University of Manchester, Manchester, UK; 5 Centre for Cancer Genetic Epidemiology, Department of Public Health and Primary Care, University of Cambridge, Cambridge, UK; 6 Centre for Cancer Genetic Epidemiology, Department of Oncology, University of Cambridge, Cambridge, UK; 7 Division of Psychology and Mental Health, School of Health Sciences, Faculty of Biology, Medicine and Health, Manchester Centre for Health Psychology, University of Manchester, Manchester, UK

**Keywords:** Health policy, Preventive medicine

## Abstract

**Background:**

National guidelines recommend English women between 30 and 49 years with moderate/high risk of breast cancer (BC) be offered screening and prevention interventions. Identification  largely relies on family history-based criteria amongst women self-presenting in primary care. We aimed to understand the preference for different potential risk assessment strategies in this population.

**Methods:**

A cross-sectional survey including a discrete choice experiment. Risk assessments were defined using six attributes, including initiation strategy, eligibility for a full/detailed risk assessment, components of the risk assessment, including multifactorial risk assessment based on the BOADICEA/CanRisk model, and sensitivity. Coefficients from conditional logistic regression were used to estimate preference for different risk assessment approaches compared to current care.

**Results:**

Participants (*n* = 893) preferred alternative approaches for risk assessments over current care, particularly where all eligible women were proactively invited and more above-average risk women were correctly identified. All but one of 21 risk assessment approaches was preferred, with the most favoured approaches having a probability of being preferred over current care >92%. Education level and ethnicity potentially impacted the strength of participant priorities.

**Conclusions:**

Offering multifactorial BC risk assessments to younger women in primary care, especially the most proactive and broad approaches, would be acceptable and preferred over current practice.

## Background

As the most common cancer worldwide, approximately 25% of cancers and 15% of cancer deaths in women are attributable to breast cancer (BC) [1], and BC incidence is projected to rise further [2]. Between 15% and 20% of BC cases occur in women younger than 50 [3]. In this age group, the global BC incidence rate has increased by 40% over the past 30 years, accompanied by a 9% rise in BC-related mortality and disability-adjusted life years [4].

The National Institute for Care Excellence (NICE) defines ‘moderate’ risk of BC as a lifetime risk from age 20 of 17–30%, or 10-year risk between ages 40 and 50 of 3–8%, and ‘high’ risk of BC as a lifetime risk from age 20 of ≥30%, or 10-year risk between ages 40 and 50 of ≥8% [5]. NICE guidelines for England state that women at moderate or high risk of BC should be offered enhanced breast imaging between ages 30 and 49 depending on risk level, alongside the offer of risk-reducing medication or surgery [5]. It is estimated that more than 250,000 women in the UK are eligible for such interventions [5] and data from clinical studies indicate that annual mammography reduces mortality from BC in this group by 12–29% [6].

The current pathway for identifying eligible women under 50 who are at moderate or high risk, however, largely relies on women self-presenting and being referred from primary care on the basis of family history alone. Referral criteria to secondary care or specialist genetics services are determined by a combination of factors, including the number of first and second-degree relatives affected by specific types of cancer, as well as the age and sex of those relatives [7]. For women who meet these criteria, secondary care or specialist genetics services are advised to perform a formal BC risk assessment using validated prediction models, which incorporate information on both unaffected relatives and relatives up to the third degree [8].

It is estimated that only a small proportion (<10%) of those at moderate or high risk are aware of their increased risk [7]. Consequently, the current pathway, initiated by women under 50 self-presenting, is estimated to miss approximately 95% of those at above-population-level risk [9] and exacerbate health inequalities [8]. There is, therefore, a clear need for a better risk assessment process for these women. In our recent systems analysis, healthcare professionals and policy stakeholders echoed a need for systemic reform that would include a more proactive approach to risk assessment [10]. Such a reform could also utilise multifactorial risk prediction tools, such as the Breast and Ovarian Analysis of Disease Incidence and Carrier Estimation Algorithm (BOADICEA) model [11, 12], which includes family history, hormonal, reproductive and lifestyle factors, and polygenic risk scores (PRS), in primary care prior to referral to secondary care or specialist genetics services. Conducting multifactorial risk assessment with all eligible women in primary care using the BOADICEA model could substantially improve identification of women at risk of developing BC over current care [9]. However, the limited capacity and expertise amongst primary care staff, the resource need, and the time commitment for women mean that a staged approach to risk assessment may remain appropriate.

It is known that people at risk of BC support risk-based approaches to guide referral decisions and incorporate genetic and non-invasive testing [13]. Specifically, women are generally positive about risk-based approaches, value the opportunity to receive risk estimates [14] and often view risk assessment as a source of reassurance and certainty [15]. A recent study further suggests that BC risk assessment would be acceptable but highlights persistent health inequalities [16] and several uncertainties, including the most effective approaches for inviting women, the acceptability of BC risk assessment amongst women, and whether proactively offering risk assessment would be cost-effective.

The present study was designed to answer some of these uncertainties and inform strategies for implementing BC risk assessment within primary care that are acceptable to the target population. The principal aim was to understand the importance of different features of such strategies from the perspective of women and the trade-offs they are willing to make. The secondary aim was to estimate the proportion of women who are likely to take up different risk assessment approaches.

## Methods

We designed and implemented a discrete choice experiment (DCE)—a method to understand which aspects of healthcare services are most valuable to the target audience [17, 18]—within a cross-sectional survey to address these aims.

### Participants and recruitment

Participants were recruited through Prolific (www.prolific.ac), an online recruitment platform for researchers in which members of the public volunteer to take part in studies. Registered female participants aged 30–49 and resident in the UK were eligible. In order for the sample to be representative of the UK adult population, we set up two surveys: one inviting people who had reported low socioeconomic status (deciles 1–3) in Prolific’s pre-screener and the other inviting people who reported middle or high socioeconomic status (deciles 4–10), with quotas for age and ethnicity within each.

Eligible participants were invited with a brief summary of the study. During the recruitment period, those interested in taking part could begin the survey, starting with the full participant information sheet and consent form, which they could download a permanent copy of. When 1000 participants had clicked through to the survey, the study was closed to further recruitment. Participants could withdraw consent for any reason at any time by using Prolific’s ‘return submission’ feature. They were paid £2.50 for completing the survey.

The target sample size (1000 participants) exceeded the sample size for DCEs calculated using the method proposed by Orme and Johnson [19, 20], which suggested a minimum of 74 participants. This large sample size was chosen to allow us to analyse the preferences of different subgroups.

### Survey design

The survey was completed online, hosted on Qualtrics (www.qualtrics.com). The survey, available as Supplementary File 1, had five main parts:

A. Information about current care and possibilities for risk assessments, with brief questions to encourage participants to consider the concepts.
B. The DCE questions, with each page containing a link to a document with a detailed explanation of the DCE levels and key terms.
C. DCE evaluation.
D. Further perspectives on risk assessments for younger women.
E. Demographic information.

### DCE design

The attributes and levels included within the DCE (survey Part B) were selected to cover the pathway for how women are currently referred to specialist breast services and options for how this might change if proactive risk assessments were implemented in primary care (Table 1). To ensure that the DCE reflected feasible potential approaches, the attributes and levels for the categorical attributes were identified from interviews and focus groups with women and key stakeholders involved in BC risk assessment [10, 21] and then refined in consultation with the wider study team. The levels of the continuous/numerical attributes were obtained from analysis of the performance of the current NICE criteria [5] and versions of the BOADICEA multifactorial risk model [11, 12] incorporating different risk factors in identifying women who developed BC within 10 years amongst 1232 women (366 BC cases within 10 years) aged under 50 years with family history and PRS data from the Generations Study (GS) [22]. Full details of the methods used in that analysis are described elsewhere [9]. Current care was based on current NICE guidelines for use in primary care [5], with women with a first- or second-degree relative with BC who present opportunistically to primary care being offered a risk assessment based on a FH of affected first- and second-degree relatives. Multifactorial risk assessment approaches were based on the BOADICEA risk model [11, 12] with different combinations of risk factors. For opportunistic approaches, it was assumed that 64% of women with a first- or second-degree FH of BC would undergo risk assessment. This was calculated based on estimates that 11% of women under age 50 have a first-degree FH of BC [23] and 9% of all women estimated to be at above-population-level risk based on multifactorial risk assessment are currently identified through referral from primary care based on family history [8]. For proactive approaches, it was assumed that 25% of women under age 50 would undergo risk assessment. This was estimated based on return of family history questionnaires in primary care in an earlier study [24] and preliminary (unpublished) data from an ongoing feasibility study of proactive BC risk assessment in primary care [25].

**Table 1:** Explanation of the attributes and levels included in the DCE.

Summary		Full explanation	
Attribute	Levels	Attribute	Levels
Risk assessment initiation	•Women contact their GP for a risk assessment^a^ •All women are invited for a risk assessment at age 35	How the breast cancer risk assessment comes about for women under the age of 50	•Women contact their GP for a risk assessment (opportunistic approach)^a^ •All women are invited for a risk assessment at age 35 (proactive approach)
Detailed risk assessment eligibility	•All women •Women with any family history of breast cancer^a^ •Only women with a family history of breast cancer – only if they have more than one relative with breast cancer or a relative diagnosed at less than age 40 years	Whether all women are offered detailed risk assessment, or only those with a certain family history of breast cancer	•All women are offered a detailed risk assessment, regardless of their family history of breast cancer •Women with a first or second degree relative with breast cancer are offered a detailed risk assessment^a^ •Only women with more than one relative with breast cancer or a relative diagnosed at less than age 40 years are offered a detailed risk assessment
Family history information	•Relatives that have had breast or ovarian cancer, and their age at diagnosis (1 to 10 min to complete)^a^ •Age or age at death for all relatives, and age at diagnosis for those with breast or ovarian cancer (15 min to 1 h to complete)	Information provided within family history of first and second-degree relatives and approximately how long this takes to complete	•Report relatives that have had breast or ovarian cancer, and the age at which they were diagnosed (1 to 10 min to complete)^a^ •Report the age or age at death for all relatives (whether they have had breast or ovarian cancer or not), and the age at which those with breast or ovarian cancer were diagnosed (15 min to 1 h to complete)
Genetic risk assessment	•No^a^ •Yes	Whether or not women will have a genetic risk assessment (through providing a saliva sample)	•No – no genetic risk assessment will be used^a^ •Yes – all women will have a genetic risk assessment
N needing risk assessment to find one case	•25 •160^a^ •300 •425	Number of women who need a risk assessment to find one woman who would develop cancer in 10 years without intervention	•25 •160^a^ •300 •425
N correctly found as above-average risk	•1 out of every 100 women who would develop cancer •3 out of every 100 women who would develop cancer^a^ •7 out of every 100 women who would develop cancer •11 out of every 100 women who would develop cancer	Out of every 100 women who would develop cancer in 10 years without intervention, number who will be found as above-average risk through the risk assessment	•1 out of every 100 women who would develop cancer •3 out of every 100 women who would develop cancer^a^ •7 out of every 100 women who would develop cancer •11 out of every 100 women who would develop cancer

^a^Used to define current care option.

It would not be feasible for participants to answer questions featuring all 147,456 potential combinations of attributes and levels. As such, an experimental design was constructed to create a smaller subset of questions while maximising the ability of the survey to identify statistically significant results based on the D-efficiency criterion (using the Stata command -dcreate-). We created 18 questions and split them into two blocks so that each participant completed nine questions, which appeared in a random order.

In each question, there were two options (labelled as ‘Option 1/2’) for proactive risk assessments plus an opt-out option defined as ‘Current care’ in line with current NICE guidelines for predicting BC risk. Participants were asked to choose between the three options in a forced-choice elicitation format with the question: ‘Which option do you think should be used in general practice to decide who should be referred to specialist services?’.

### Additional survey questions

Before the DCE questions, participants were asked to rate how comfortable they would be providing a detailed family history, completing a questionnaire about BC risk factors, and providing a saliva sample for PRS on a 5-point Likert scale (Part A).

After the DCE questions, participants reported how easy or difficult they found the DCE and reflected on the approach they took to making their selections (Part C). They then answered additional multiple-choice questions about risk assessments (the age at which they think they should be offered, their preferred mode of invitation, locations they would be willing to have a breast density assessment, and opinions about their overall acceptability based on the Theoretical Framework of Acceptability [26, 27]) (Part D). Finally, participants provided demographic information, family history of cancer, and perceived likelihood and worry about developing cancer in the future (Part E).

### Patient and public involvement and engagement (PPIE)

We invited members of the study PPIE group (nine women who were aged 30–49, had no history of BC, lived in a low socioeconomic area, and collectively represented multiple ethnic groups) to review the survey outline, participant information sheet, and consent form. They were asked to comment on clarity, comprehensibility, and relevance. Particular attention was requested for the survey background information and an example DCE question, including whether sufficient information was provided to understand the DCE questions and whether any content could be streamlined.

Two PPIE members provided feedback via email and annotated documents, which was subsequently incorporated into the study materials. They were reimbursed for their time contributing to the research activities.

### Analysis

Any participants with indicators of poor attention (defined as completing the total survey in <5 min or always selecting option 1 or always selecting 2 within the DCE) were excluded prior to the analysis.

Participants’ characteristics and responses to the additional questions about their perspectives towards risk assessments were summarised using descriptive statistics. Missing demographic data were replaced with data provided by Prolific, where applicable.

DCE data were analysed using conditional logistic regression. The model that best fit the data was identified by examining the Akaike and Bayesian information criteria (AIC and BIC) of increasingly complex models: (1) a fixed effects conditional logistic regression model; (2) model 1 with and without constants for options 1 and 2 to examine tendencies for selecting the left-hand or right-hand option presented in each DCE task regardless of the attributes; (3) the previous best model with each continuous attribute effects-coded to check for co-linearity; (4) the previous best model with each attribute with evidence of non-linearity transformed (quadratic, logarithmic and piecewise). Additionally, the relative importance of each attribute (RAI) was calculated as the difference in the logistic regression coefficients multiplied by the most- and least-preferred level and presented as a percentage [28].

We also calculated the probability that the population would take up multiple different risk assessment options compared to current care, which was set to 50% probability of preference.

Lastly, we investigated whether priorities for risk assessments varied according to key demographic characteristics. This was done by comparing the RAI between each binary subgroup (age, ethnicity, education, socioeconomic status, and family history of BC; defined below) using Chi-squared tests.

All analyses were performed using Stata 15 and Microsoft Excel.

## Results

### Responses and participant characteristics

Survey data were collected between 29 and 31 July 2025. At that time, there were 15,193 eligible participants active on Prolific; 997 began the survey and 927 completed it. 55 responses were excluded due to indications of poor attention; therefore, a total of 893 responses were analysed. Median time to complete the survey was 11 min 12 s (interquartile range 8 min 26 s to 15 min 10 s).

Table 2 shows the characteristics of the participants. Reflecting the recruitment strategy, 53% were under 40 years of age, and the ethnicity distribution was comparable to the UK population, including 81% white participants. 63% of participants had an undergraduate or higher degree and 81% considered their socioeconomic decile to be mid or high. Only 20 participants (2%) had had any type of cancer themselves, while 31% had a family history of cancer and 54% knew a close friend or other family member with a history of cancer. 45% considered that they were slightly likely (18.5%) or extremely or moderately likely (26.3%) to develop cancer in the next 10 years but only 19% thought about this often or a lot.

**Table 2:** Participants’ demographic characteristics, familiarity with cancer and worry about cancer.

	*N*	(%)
Total	893	100
Demographics		
Age range		
30–34	243	27.2
35–39	231	25.9
40–44	254	28.4
45–49	165	18.5
Simplified ethnicity		
Asian or Asian British	70	7.8
Black, Black British, Caribbean or African	35	3.9
Mixed or multiple ethnic groups	63	7.1
White	720	80.6
Other ethnic group	5	0.6
Education		
No education or completed GCSEs, O Levels or equivalent	97	10.9
Completed A Levels, further education or equivalent	232	26.0
Completed a Bachelor’s/undergraduate degree	382	42.8
Completed a Master’s degree, PhD or other postgraduate qualification	182	20.4
Perceived position on socioeconomic ladder		
1–3 (low)	170	19.0
4–7	685	76.7
8–10 (high)	38	4.3
Familiarity with and worry about cancer		
Personal history of cancer		
Yes	20	2.2
No	871	97.5
Prefer not to say or missing (not reported)	2	0.2
Family history of cancer		
Yes	277	31.0
No	596	66.7
Prefer not to say or don’t know (not reported)	20	2.2
Significant other’s history of cancer		
Yes	479	53.6
No	394	44.1
Prefer not to say or don’t know (not reported)	20	2.2
Perceived likelihood of developing cancer in the next 10 years		
Extremely or moderately likely	235	26.3
Slightly likely	165	18.5
Neither likely nor unlikely	251	28.1
Slightly unlikely	75	8.4
Extremely or moderately unlikely	132	14.8
Prefer not to say or missing (not reported)	35	3.9
Frequency of thinking about chance of getting cancer in the past month		
Often or a lot	165	18.5
Not at all, rarely or sometimes	722	80.9
Prefer not to say or missing (not reported)	6	0.7

### Model selection

Following the model selection process, described in Table S1, model 1 was selected because the AIC and, in particular, BIC were not sufficiently lower to justify the increased complexity of the other models. In this analysis, categorical attributes were effects-coded, and continuous attributes were treated as continuous. Positive coefficients indicate preferences for the level indicated and vice versa, and the alternative specific constant (ASC) indicates preferences for current care.

### Priorities for risk assessments

Primary results of the DCE are presented in Table 3. Overall, participants preferred the alternative approaches for risk assessments over current care, indicated by the negative ASC coefficient, −0.65 (95% confidence interval −0.74 to −0.55). Preferences were for women to be proactively invited to risk assessments rather than waiting for self-presentation in primary care. Participants also preferred less stringent detailed risk assessment eligibility, with everyone eligible the most-favoured option and having any family history preferred over needing a strong family history. For the detail of family history information provided within the detailed risk assessment, describing those affected by breast and ovarian cancer only was favoured over a longer, full family history including non-cases. Participants were also positive about having a PRS measured. Fewer risk assessments to find one woman who would develop cancer was considered better, indicated by the negative coefficient. Options that are better at finding women who would be more likely to develop cancer without intervention were also considered better, indicated by the positive coefficient. All coefficients were statistically significant (*p* < 0.001).

**Table 3:** Conditional logistic regression results.

	Coefficient (95% CI)	RAI (%)
Current care (ASC)	-0.65 (-0.74 to -0.55)***	
Risk assessment initiation		
Opportunistic	−0.59 (−0.54 to −0.64)***	29.4
Proactive	0.59 (0.54 to 0.64)***	
Detailed risk assessment eligibility		
All	0.29 (0.22 to 0.35)***	18.6
Any FH	0.17 (0.05 to 0.30)**	
FH in >1 relative or <40 yrs	−0.46 (−0.53 to −0.38)***	
Family history information		
Affected FH	0.14 (0.19 to 0.09)***	7.0
Full FH	−0.14 (−0.19 to −0.09)***	
Genetic risk assessment		
No	−0.34 (−0.29 to −0.40)***	17.3
Yes	0.34 (0.29 to 0.40)***	
N needing risk assessment to find one case		
	−0.0005 (−0.0007 to −0.0003)***	4.7
N correctly found as above-average risk		
	0.09 (0.08 to 0.10)***	23.0

Number of participants: 893, number of observations: 24,111, pseudo *R*^2^: 0.1986. ^*^p ≤ 0.05; ^**^p ≤ 0.01; ^***^p ≤ 0.001.

*ASC* alternative specific constant, *CI* confidence interval, *FH* family history (of breast or ovarian cancer), *N* number of women, *RAI* relative attribute importance.

The RAI shows that how risk assessments are initiated was participants’ most important attribute when choosing between options (RAI 29%). The number of women who would develop cancer correctly identified to be at above-average risk; who is offered detailed risk assessment; and whether a genetic risk assessment is included were also similarly important (RAI 19–23%). Details of family history and number of women who need a risk assessment to find one who would develop cancer were least important.

### Probability of preferring different risk assessment options compared to current care

Table 4 shows the modelled probability that participants would prefer 21 different risk assessment options compared to the current care reference. All risk assessments, except the opportunistic model 7 (with the least-preferred levels for all categorical attributes and low preference values for continuous attributes), were more likely to be preferred than current care (probability of preference >50%). All proactive strategies had a probability of preference >69% and were preferred over the equivalent opportunistic strategy in each case despite correctly finding fewer women at above-average risk. Notably, this includes using the existing NICE criteria (proactive model 1), which had a probability of preference of 84% over current practice (opportunistic use of the NICE criteria). Conversely, offering detailed risk assessment only to those with a strong FH (FH in >1 relative or <40 years) resulted in a substantial (9–25%) reduction in preference over the equivalent strategy in which all women or those with any FH were offered a detailed risk assessment; all strategies, both opportunistic and proactive, in which the risk assessment was offered to all women or those with any FH had a probability of preference >74%. The most preferred strategies (probability of preference >92%) were those that included proactive risk assessment incorporating PRS for either all women or those with any FH (proactive models 2,3, 8 and 9).

**Table 4:** Probability of preferring different risk assessment options compared to current care.

	Model	Detailed risk assessment eligibility	Family history information	Genetic risk assessment	*N* needing risk assessment to find one case	*N* correctly found as above-average risk	Preference (%)
	Opportunistic assuming 64% of those with a FH (7% of women under aged 50) present to primary care for a risk assessment						
1	Current care (NICE)	Any FH	Affected FH	No	160	3	Reference (50)
3	BOADICEA	Any FH	Full FH	Yes	45	10	86
4	BOADICEA	FH in >1 relative or <40 yrs	Full FH	Yes	25	2	61
6	BOADICEA	Any FH	Full FH	No	46	10	74
7	BOADICEA	FH in >1 relative or <40 yrs	Full FH	No	24	3	44
9	BOADICEA	Any FH	Affected FH	Yes	44	10	88
10	BOADICEA	FH in >1 relative or <40 yrs	Affected FH	Yes	25	2	68
12	BOADICEA	Any FH	Affected FH	No	43	10	79
13	BOADICEA	FH in >1 relative or <40 yrs	Affected FH	No	24	3	51
	Proactive assuming 25% of women under age 50 take up risk assessment						
1	NICE	Any FH	Affected FH	No	160	1	84
2	BOADICEA	All	Full FH	Yes	185	9	95
3	BOADICEA	Any FH	Full FH	Yes	45	4	92
4	BOADICEA	FH in >1 relative or <40 yrs	Full FH	Yes	25	1	82
5	BOADICEA	All	Full FH	No	350	5	85
6	BOADICEA	Any FH	Full FH	No	45	4	85
7	BOADICEA	FH in >1 relative or <40 yrs	Full FH	No	24	1	69
8	BOADICEA	All	Affected FH	Yes	173	9	96
9	BOADICEA	Any FH	Affected FH	Yes	44	4	94
10	BOADICEA	FH in >1 relative or <40 yrs	Affected FH	Yes	25	1	85
11	BOADICEA	All	Affected FH	No	333	5	89
12	BOADICEA	Any FH	Affected FH	No	43	4	88
13	BOADICEA	FH in >1 relative or <40 yrs	Affected FH	No	24	1	75

BOADICEA: Breast and Ovarian Analysis of Disease Incidence and Carrier Estimation Algorithm; FH: family history (of breast or ovarian cancer); N: number of women; NICE: National Institute for Health and Care Excellence. Models numbered as in Frost et al [9]. Labelling equivalence: risk assessment initiation – proactive = proactive, opportunistic = non-proactive; detailed risk assessment eligibility – all = filter 0, any FH = filter 1, FH in > 1 relative or <40 yrs = filter 2; family history information – affected FH = -ive FH false, full FH = -ive FH true; genetic risk assessment – yes = PRS true, no = PRS false; N needing risk assessment to find one case = N assessed for one case; N correctly found as above-average risk = sensitivity.

### Differences in priorities according to participant characteristics

**Fig. 1:**
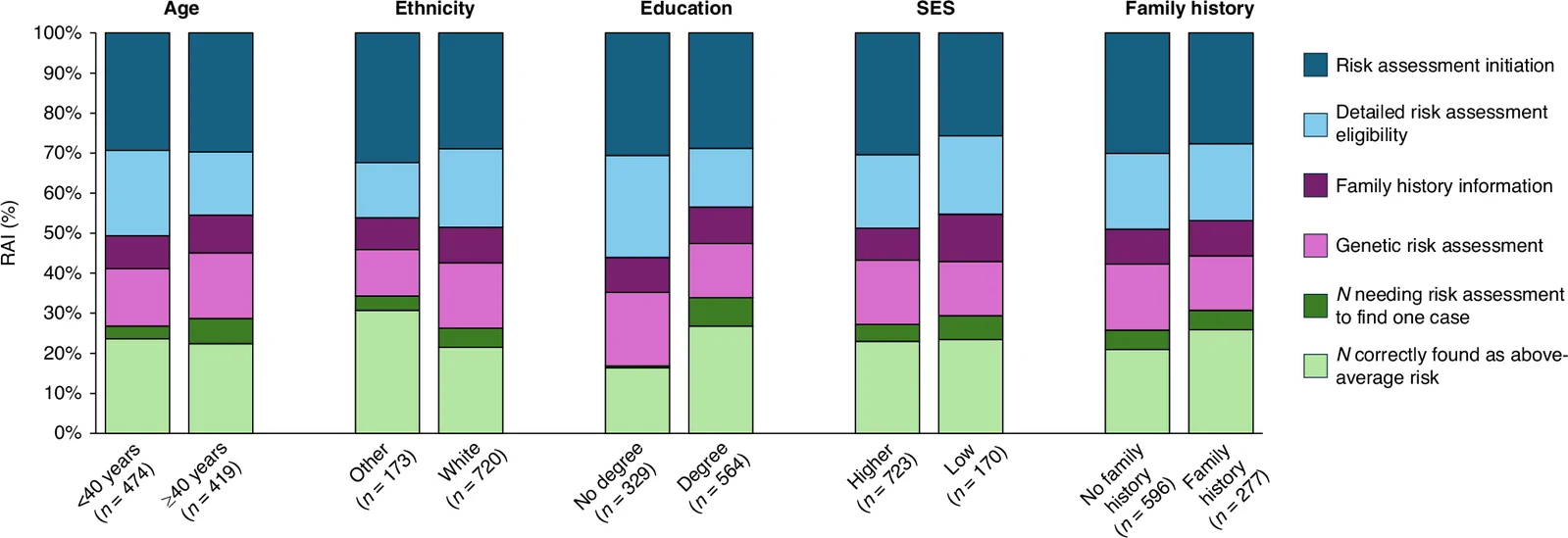
Relative importance of each attribute by binary participant characteristics. FH: family history (of breast and/or ovarian cancer); N: number of women; n: number of participants; RAI: relative attribute importance; SES: perceived position on socioeconomic ladder.

Figure 1 shows the RAI for each subgroup based on the conditional logistic regression results (Table S2). There were no differences across any of the subgroups with regard to the importance of how risk assessments are initiated, detail of family history information, and whether PRS is included, and no differences across any of the attributes with age or SES. The largest difference was between the models for those with, versus without, a degree-level education (p for the overall model <0.0001). This was particularly with regard to detailed risk assessment eligibility, with participants without a degree considering this attribute more important (RAI 25.5% versus 14.7% for participants with a degree), and the number of women who need a risk assessment to find one case and who would be found as above-average risk, with participants with a degree considering these to be much more important. In addition, White participants’ views differed to those of other ethnic groups (p for the overall model 0.003), driven by the greater importance of eligibility for a detailed risk assessment and lower importance of number of women who need a risk assessment to find one case to White participants (RAI 19.6% versus 13.7% and 21.5% versus 30.8% for White versus other ethnicity participants, respectively). No differences were seen with the overall model (*p* = 0.336), the order of importance of the attributes, or for importance of how risk assessments are initiated, detailed risk assessment eligibility, detail of family history information, whether PRS is included, or the number needing risk assessment to find one case for the models for those with, versus without, a family history. Those women with a family history, however, considered the number correctly found as above-average risk more important than those without a family history (RAI 25.8% versus 21.0%).

### DCE evaluation

Most participants (60%) reported that it was hard to choose between options when completing the DCE (Table S3). Half said that they took just one or two attributes into account, while 42% took three or four attributes into account. When ranking the attributes, the most important were who is offered a detailed risk assessment and the number of women who would develop cancer who are found as above-average risk. These were followed by when risk assessment is initiated, then by the number of women who need a risk assessment to find one who would develop cancer. The least important attributes were family history information and genetic risk assessment, which were ranked lowest and least often considered.

### Further views towards risk assessments

Having read brief notes about what detailed risk assessments could involve prior to the DCE, almost all participants reported that they would feel comfortable or extremely comfortable completing each aspect (Table 5, and Fig. S1). They were most positive towards completing a risk factors questionnaire (only 2% would be uncomfortable), followed by PRS and a detailed family history (each 4%).

**Table 5:** Additional participant perspectives on breast cancer risk assessments for younger women.

	*N*	(%)
Total	893	100
Comfort with aspects of detailed risk assessments (n/% comfortable)^a^		
Giving a detailed family history of affected and unaffected relatives	797	89.2
Completing a risk factors questionnaire	849	95.1
Completing a genetic breast cancer risk assessment	802	89.8
Perspectives towards additional aspects of risk assessments		
At what age do you think risk assessments should be offered?		
30 years	395	44.2
35 years	384	43.0
40 years	81	9.1
45 years	7	0.8
50 years	8	0.9
None^b^	18	2.0
What do you think the best way to invite women would be?		
Letter from the NHS/GP practice posted to the home address	333	37.3
Text message from the NHS/GP practice	224	25.1
Phone call from the GP practice	28	3.1
NHS app notification	30	3.4
At the same time as invitations for cervical screening and/or NHS Health Checks	250	28.0
Other/multiple	23	2.6
None^a^	5	0.6
Where would you be willing to have breast density assessment as part of a risk assessment?		
Hospital	684	76.6
Mobile or community unit	669	74.9
GP practice	750	84.0
None^c^	19	2.1
Acceptability of breast cancer risk assessments (n/% in agreement)^a^		
“Overall, I find risk assessments for breast cancer for younger women to be acceptable”	732	82.0
“It makes sense to me that risk assessments for younger women could help find breast cancer early”	863	96.6
“Risk assessments for younger women could lead to improvements in breast cancer survival”	863	96.6
“Risk assessments for younger women would be a good use of NHS funding”	799	89.5
“Offering risk assessments for younger women is fair…		
for society as a whole”	817	91.5
for younger women”	851	95.3
for older women and men”	699	78.3

^a^Binary response for extremely comfortable/acceptable and comfortable/acceptable versus neither comfortable/acceptable, uncomfortable/unacceptable and extremely uncomfortable/unacceptable.

^b^“None – I don’t think breast cancer risk assessments should be offered to younger women.”

^c^“None – I would not be willing to have a breast density assessment.”

In additional questions answered after the DCE, 87% of participants favoured such risk assessments being introduced for women aged 30 or 35 (Table 5). A postal letter was considered the preferred method of invitation by 38% of participants, followed by at other appointments (28%) or a text message (25%); phone calls and NHS app notifications were disliked. Almost all participants (98%) suggested they would be happy to have a breast density assessment as part of a risk assessment. More than 75% of participants were happy to attend either a hospital, mobile/community unit or GP practice, with GP practice the preferred location for a breast density assessment.

Overall, risk assessments were acceptable to 82% of participants (Table 5, and Fig. S2). They were considered to facilitate early diagnosis and lead to improvements in BC survival, and be a good use of NHS funding. The majority thought they were fair, although they were most clearly fair to younger women (95%) and least fair to older women and men (78%).

## Discussion

The findings of this study show that the public strongly support the concept of proactive and multifactorial BC risk assessments for younger women in primary care over the current approach which largely relies on women self-presenting with concerns about a family history. Whilst almost any of the alternative approaches considered in this study would be supported, participant priorities were accuracy and inclusivity.

The importance of accuracy, here represented by sensitivity, was an expected finding. It is consistent with public desire for assurance of risk assessment accuracy in order to accept risk-stratified breast screening [29, 30] and the prioritization of sensitivity in novel technologies for cancer risk assessment [13].

Of greater significance was the importance of inclusivity, with strong preferences for having proactive invitations rather than relying on women self-presenting if they have a family history of BC, as well as lower requirements for family history so that more women are eligible for a detailed risk assessment. Within the range of levels used within this study, these two attributes drive many of the preferences. For example, offering risk assessment based on current NICE guidelines proactively (proactive model 1) is preferred over opportunistic assessment (84% probability) despite that approach missing more women who would develop cancer without intervention. Offering multifactorial risk assessment without PRS but restricting eligibility to women with more than one relative with breast cancer or a relative diagnosed at less than age 40 years (opportunistic model 7) is also less preferred (44% probability) than current practice despite identifying the same number of women who would develop cancer without intervention but with nearly seven times fewer women needing to be risk assessed (*n* = 24 vs *n* = 164). This highlights how the public are more interested in equity of access than equity of outcomes and are not concerned with resource use. It may also relate to wider public concern about the fairness of including family history in policies for access to healthcare. In previous studies, participants have worried that people may ‘miss out’ because they are unaware of family history of cancer due to, for example, adoption or relatives’ early death from other causes [31]. Concerns around eligibility based on family history may also be particularly pertinent in certain ethnic or cultural communities in which diagnoses are not openly shared within the family.

Additionally, both the DCE and survey suggest a high level of comfort with each of the elements of a full risk assessment. Participants preferred options in which PRS were included and 90% reported they would be comfortable completing a genetic test. This may reflect a view that PRS is considered a consistent and objective measure of risk, particularly when compared to family history [32]. 89% would also be comfortable giving a family history of both affected and unaffected relatives, albeit with a preference for a shorter family history of affected relatives only. Likewise, almost all participants would be comfortable completing the risk factors questionnaire, which was framed as always being part of the full risk assessment, and an assessment of breast density if this were to be included in the future.

We did not identify major differences in participants’ perspectives towards risk assessments by their demographic characteristics. In particular, there were no differences across the subgroups with regard to the importance of how risk assessments are initiated, detail of family history information, and whether PRS is included, and no differences were observed by family history of cancer status. The differences we did observe were in priorities according to education level and ethnicity, with women with higher education levels and ethnicity other than white placing greater importance on accuracy than eligibility requirements.

To our knowledge, this is the first study to quantify the relative importance of different approaches to eligibility and invitation for BC risk assessment amongst young women. It is important, however, to consider how recruitment and selection bias may have impacted the findings. While recruiting through an online recruitment platform enabled us to recruit a cohort representative of the UK with respect to age, ethnicity and socioeconomic status, which would not have been possible if we had recruited through primary care, people registered with the recruitment agency are inevitably engaged with sharing their opinions and have access to the internet. The sample may also not have been representative with respect to other characteristics. For example, the proportion who reported a first- or second-degree family history of BC was higher amongst participants than in the general population. 45% also considered they were likely to develop cancer in the next 10 years. While this is comparable to other studies using similar recruitment methods [33] and it is well reported that the general public overestimate their risk [34], it is not known if this reflects the general population or if the participants of this study had more concerns about developing BC. Additionally, those who accepted the invitation to the survey may have greater interest in BC prevention and early diagnosis and healthcare services research, corresponding to more positive views towards the introduction of risk assessments for BC than are held by non-participants. Our findings from subgroup analyses suggest that any differences, particularly with family history, were likely to be small, but we cannot rule out an impact of recruitment and selection bias. Also, we aimed for participants to be sufficiently informed about the suggested BC risk assessments and how this differed from the current system through background text, the questions about their comfort in taking part in different elements of risk assessments and by providing a reminder of the key concepts available alongside each DCE question. How well participants understood the study and made use of the resources is, however, unknown. While based on data from a cohort of women with BC outcomes and weighted to the UK population, the estimates of the number of women needing a risk assessment to identify one women who would go on to develop BC within 10 years without intervention, and the number of women who would go on to develop BC within 10 years without intervention who would be identified at above-population level risk are also based only on white women and the numbers of women with more than one relative with breast cancer or a relative diagnosed at less than age 40 years are small. We also had to estimate the proportion of women undergoing risk assessment for both opportunistic and proactive approaches and have not accounted for women referred for risk assessment from surgical services after presenting with a breast complaint.

Ultimately, the decision around when and where to offer risk assessment and which risk factors to include will need to consider the practical, resource, expertise, and cost implications of data collection and risk assessment and the potential benefits and harms associated with accurate and inaccurate classification of women alongside the views of the public. Our findings clearly show that there is public enthusiasm and support for proactive multifactorial BC risk assessment in primary care. Almost all approaches of either proactive or multifactorial risk assessment would be preferred by the public over current care and so would be acceptable options. The importance that the public place on equity of access over outcomes or resource use will, however, be important for researchers and policy makers planning and evaluating potential approaches and highlights the importance of being able to clearly justify any decisions not to adopt a universal proactive approach in communication materials.

## Supplementary information

Supplementary File 1

Supplementary File 2

Reporting checklist

## Data Availability

Data are available in a public, open access repository. All the individual, de-identified participant data collected as part of the study are available on the University of Cambridge repository (https://doi.org/10.17863/CAM.121965). The study protocol, study documents (participant information sheet and consent form), survey and data dictionary are available on the repository. Data are available with no end date without restrictions.
