# Supplementary material for: Priorities for breast cancer risk assessment in UK women under age 50: a survey and discrete choice experiment
Source: Br J Cancer. 2026 Aug 4;135(9):1430–8. doi: 10.1038/s41416-026-03546-3 (PMC13675216; doi:10.1038/s41416-026-03546-3)
Supplement: Supplementary file 1 [file 41416_2026_3546_MOESM1_ESM.pdf]

PREVENT BREAST
DCE SURVEY OUTLINE

[Note: shaded text and notes were not seen by participants]

Part A. Perspectives on key attributes

Context

What this study is about

✓ This study considers women without symptoms of breast cancer.

✗ It does not consider what happens when people have symptoms. People with symptoms should speak to their GP about their concerns.

✓ This study is about breast cancer risk assessment. A risk assessment is used to calculate how likely someone is develop breast cancer in the future.

✗ It is not about screening or diagnosis to detect breast cancer.

✓ This study is about breast cancer risk assessment for women who are not yet old enough to be invited for standard breast screening.

✗ It is not about breast cancer screening for women between the ages of 50 and 71. These women will be invited for NHS breast screening as usual.

Current care

At the moment in England, anyone under the age of 50 who is concerned about their risk of breast cancer can make an appointment with their GP. The GP will ask whether any family members have had breast cancer. Based on this information, they may refer women to **specialist services** (e.g., clinics specialising in genetics or breast health).

The specialist clinicians check the information the women have already provided and then potentially ask for more information on their family, including family members not affected by cancer, and/or do other tests, such as genetic tests or assessment of breast density, to inform the risk assessment.

Depending on the results of the risk assessment, women might be offered treatment to lower their chances of developing breast cancer (like risk-reducing medication or risk-reducing surgery), and/or additional breast screening.

Changes suggested in this study

Family history, genetics and other risk factors can all contribute to risk of developing breast cancer. Instead of GPs only asking about family members with breast cancer and waiting for women to come to them with concerns, these other risk factors could also be assessed in primary care (through the GP practice) to help inform who should be referred to **specialist services** and women could be invited to have a risk assessment.

Including all these risk factors would mean that the initial risk assessment done through the GP practice would be more accurate. It would take longer though and require more information from all women, not just those who are referred to specialist services.

Inviting all women to have a risk assessment would likely find more women with an increased risk of breast cancer but would also mean doing lots of risk assessments on women who are not at increased risk.

In this study, we want to understand what aspects of risk assessment women would like to be done in primary care (through general practice) and whether all women should be invited to do a risk assessment rather than waiting for women to contact their GP.

[Note: Page break]

Detailed risk assessments

There are three possible parts of more detailed risk assessments to consider.

1. Family history

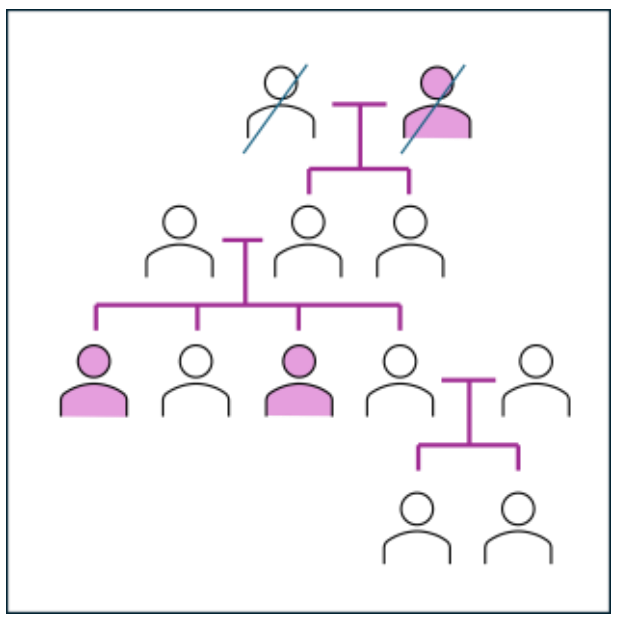

Giving a family history usually involves considering **first and second-degree relatives** – parents and children, grandparents and grandchildren, brothers and sisters, and aunts/uncles and nieces/nephews.

It can involve either reporting simply whether anyone has been **affected** (whether or not anyone has had breast and/or ovarian cancer) or a **detailed family history of both affected and unaffected** relatives (all the relatives you have, their age/age when they died, whether each has had breast or ovarian cancer, and when they were diagnosed). Some people might want to contact their relatives for information to help them complete it accurately.

1. How comfortable would you be giving a **detailed family history** of **both affected and unaffected** relatives?
    - ○ Extremely uncomfortable
    - ○ Uncomfortable
    - ○ Neither uncomfortable nor comfortable
    - ○ Comfortable

- Extremely comfortable

2. Risk factors questionnaire

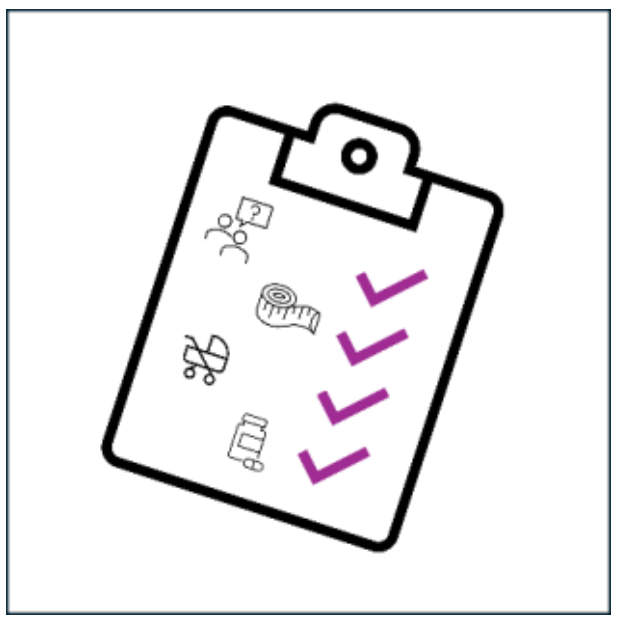

Providing information about breast cancer risk factors involves completing a questionnaire including information about:

Your height and weight
Number of children you have had, their sex and at what age
Age at when you started your periods and, if relevant, when you went through the menopause
Use of oral contraceptives and hormone replacement therapy (HRT)
Alcohol intake
Ethnicity and whether you have Ashkenazi Jewish ancestry
Whether you or other family members have had other rare cancers

2. How comfortable would you be completing a **risk factors questionnaire**?
   - Extremely uncomfortable
   - Uncomfortable
   - Neither uncomfortable nor comfortable
   - Comfortable
   - Extremely comfortable

3. Genetic risk

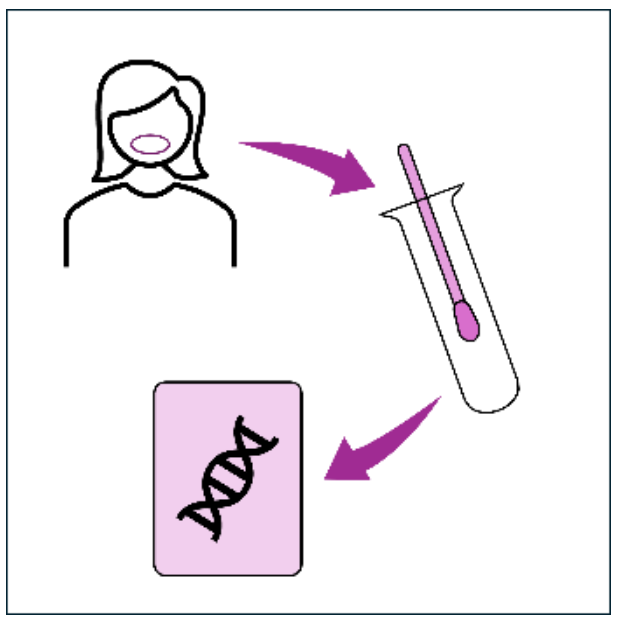

To do a genetic test, you would need to provide a saliva sample or cheek swab. This would allow doctors/scientists to estimate your risk of breast cancer based on your genes. Specifically, this is called on "polygenic risk", and it considers tiny variations in lots of genes, some of which increase risk and others that protect against breast cancer.

3. How comfortable would you be completing a **genetic breast cancer risk assessment**?
   - Extremely uncomfortable
   - Uncomfortable
   - Neither uncomfortable nor comfortable
   - Comfortable
   - Extremely comfortable

[Note: Page break]

Part B. DCE

We will now present you with a set of nine questions with two different options for risk assessments in each, plus current care. Each time the features of the risk assessments (in the rows of the table) will change.

For each question, please select the option that you think should be used in general practice to decide who should be referred to specialist breast services.

These questions are required, so you won't be able to skip any of them. Sometimes the answer might seem obvious to you. Other times it might be harder to work out what you think is best. **The questions are about your opinion so there is not a correct answer.** Depending on the size of your computer screen, you might find it helpful to zoom in to see the table.

[Note: Page break]

[Note: Example DCE question]

Question 1 of 9

Q1	Option 1	Option 2	Current care
**Risk assessment initiation**	Women contact their GP for a risk assessment	All women are invited for a risk assessment at age 35	Women contact their GP for a risk assessment
**Who is offered detailed risk assessment**	All women	Only women with a family history of breast cancer – only if they have more than one relative with breast cancer or a relative diagnosed at less than age 40	Women with any family history of breast cancer
Within the detailed risk assessment, all women will complete the additional **risk factors questionnaire** and **family history**, and some will have a **genetic risk assessment**:			
**Family history information** provided, and **time needed**	Age or age at death for all relatives, and age at diagnosis for those with breast or ovarian cancer (15 minutes to 1 hour)	Relatives that have had breast or ovarian cancer, and their age at diagnosis (1 to 10 minutes)	Relatives that have had breast or ovarian cancer, and their age at diagnosis (1 to 10 minutes)
Offering **genetic risk assessment**	No	Yes	No
Number of women who **need a risk assessment** to **find one** who would develop cancer	160	25	160
Number of women who **would develop cancer** who will be **found as above-average risk through the risk assessment**	11 out of every 100 women who would develop cancer	7 out of every 100 women who would develop cancer	3 out of every 100 women who would develop cancer

Click *__here__* [Note: See end of document] *to open a more detailed explanation of the important words and ideas in this table.*

Which option do you think should be used in general practice to decide who should be referred to specialist services?

- **Option 1**
- **Option 2**
- **Current care**

[Note: Page break]

Part C. DCE evaluation

Evaluation

1. In general, how easy or difficult did you find choosing between the different options?
    - Very easy
    - Easy
    - Slightly easy
    - Slightly difficult
    - Difficult
    - Very difficult

2. Please tell us a few words why you found choosing between the different programmes easy or difficult.
    - [Free text]

3. When choosing between Option 1, Option 2, and current care, which of the following factors were important in your decision? Please select all that apply.
    - How the risk assessment is initiated
    - Whether all women are offered detailed risk assessment, or only those with a family history of breast cancer
    - Information provided within family history of first and second-degree relatives (and approximately how long this takes)
    - Whether or not women will have a genetic risk assessment (through providing a saliva sample)
    - Number of women who need to be referred to specialist services to find one at above average risk of breast cancer (i.e. are correctly referred)
    - Number of women who are at above average risk of breast cancer but not referred to specialist services (i.e. are missed)

    [Selected responses carried forward to next question]

[Note: Page break]

4. Please rank the following factors in order of their importance to you when choosing between the different options, with 1 = most important.
    - [Populated with responses selected from the previous question]

[Note: Page break]

Part D. Further views on risk assessments

Further views on risk assessments

1. In the questions you have just answered, we said that all women could be invited for a breast cancer risk assessment at age 35. At what age do you think risk assessments should be offered?
    - 30 years
    - 35 years
    - 40 years
    - 45 years
    - 50 years
    - None – I don't think breast cancer risk assessments should be offered to younger women
2. What do you think the best way to invite women would be?
    - Letter from the NHS/GP practice posted to the home address
    - Text message from the NHS/GP practice
    - Phone call from the GP practice
    - NHS app notification
    - At the same time as invitations for cervical screening and/or NHS Health Checks
    - Other – please describe _____
    - None – I don't think breast cancer risk assessments should be offered to younger women
3. Breast density can also help doctors to estimate risk of breast cancer alongside family history, risk factor questionnaires and genetics. This could be done using a mammogram (an x-ray of the breast, like is used for breast cancer screening), ultrasound, or other types of scan as technologies develop. This test would <u>not</u> be designed to detect breast cancer. Where, if any, of the following locations would you be willing to have breast density assessment as part of a risk assessment? Select all that apply.
    - Hospital
    - Mobile or community unit
    - GP practice
    - None – I would not be willing to have a breast density assessment as part of a breast cancer risk assessment through general practice
4. Lastly, we want to understand your thoughts and opinions about the overall acceptability of breast cancer risk assessments for younger women (aged 30-50) in general practice. How much do you agree or disagree with the following statements? [strongly disagree, disagree, neither agree nor disagree, agree, strongly agree] [presented in matrix]
    - Overall, I find risk assessments for breast cancer for younger women to be acceptable

- It makes sense to me that risk assessments for younger women could help find breast cancer early
- Risk assessments for younger women could lead to improvements in breast cancer survival
- Risk assessments for younger women would be a good use of NHS funding
- Offering risk assessments for younger women is fair...
    - ...for society as a whole
    - ...for younger women
    - ...for older women and men

[Note: Page break]

Part E. Demographics

Demographic information

In this last section, please answer a few questions about yourself. These questions allow us to make sure we include people from a range of backgrounds and see if different groups of people have different views. We will not be able to identify you from your answers.

1. How old are you?
    - ___ years

2. Which option best describes your ethnic group or background?
    - White
        - English, Welsh, Scottish, Northern Irish or British
        - Irish
        - Gypsy or Irish Traveller
        - Roma
        - Any other White background, please describe__________
    - Mixed or Multiple ethnic groups
        - White and Black Caribbean
        - White and Black African
        - White and Asian
        - Any other Mixed or Multiple ethnic background, please describe__________
    - Asian or Asian British
        - Indian
        - Pakistani
        - Bangladeshi
        - Chinese
        - Any other Asian background, please describe__________
    - Black, Black British, Caribbean or African
        - Caribbean
        - African, please describe__________

        - Any other Black, Black British or Caribbean background, please describe__________
    - Other ethnic group
        - Arab
        - Jewish
        - Any other ethnic group, please describe__________
    - Prefer not to say

3. What is your highest education level?
    - Finished school at or before the age of 15 years
    - Completed GCSEs, O Levels or equivalent
    - Completed A Levels or equivalent
    - Completed further education but not a degree
    - Completed a Bachelor's degree
    - Completed a Master's degree, PhD or other postgraduate qualification
    - Other (please describe)

4. Where would you put yourself on the socio-economic ladder?
   Think of this ladder as representing where people stand in our society. At the top of the ladder (10) are people better off with most education, money and desirable jobs. At the bottom of the ladder (1) are people worse off with least education, money and undesirable jobs.
    - 10 – 1

[Note: Page break]

5. Have you ever had cancer?
    - Yes
    - No
    - Prefer not to say

6. Have your parents or any brothers or sisters ever had cancer?
    - Yes
    - No
    - Don't know
    - Prefer not to say

7. Has anyone close to you (e.g. a partner or close friend) ever had cancer?
    - Yes
    - No
    - Don't know
    - Prefer not to say

8. How likely do you think is it that you will get cancer at some point in the next 10 years?
    - Extremely likely

- Moderately likely
- Slightly likely
- Neither likely nor unlikely
- Slightly unlikely
- Moderately unlikely
- Extremely unlikely
- Prefer not to say

9. During the past month, how often have you thought about your own chances of getting cancer?
    - Not at all
    - Rarely
    - Sometimes
    - Often
    - A lot
    - Prefer not to say

[Note: End of survey]

Further explanation of important concepts

More detailed explanation of the factors used in the main tables

	**Explanation**	**Possible options**
**Risk assessment initiation**	How the breast cancer risk assessment comes about for women under the age of 50	• Women contact their GP for a risk assessment (opportunistic approach)<br>• All women are invited for a risk assessment at age 35 (proactive approach)
Who is **offered detailed risk assessment**	Whether all women are offered detailed risk assessment, or only those with a family history of breast cancer	• All women are offered a detailed risk assessment, regardless of their family history of breast cancer<br>• Women with a first or second degree relative with breast cancer are offered a detailed risk assessment<br>• Only women with more than one relative with breast cancer or a relative diagnosed at less than age 40are offered a detailed risk assessment
Within the detailed risk assessment, all women will complete the **additional risk factors questionnaire** and **family history**, and some will have a **genetic risk assessment:**		
**Family history information** provided, and **time needed**	Information provided within family history of first and second-degree relatives (and approximately how long this takes)	• Report relatives that have had breast or ovarian cancer, and the age at which they were diagnosed (1 to 10 minutes)<br>• Report the age or age at death for all relatives (whether they have had breast or ovarian cancer or not), and the age at which those with breast or ovarian cancer were diagnosed (15 minutes to 1 hour)
Offering **genetic risk assessment**	Whether or not women will have a genetic risk assessment (through providing a saliva sample)	• No – no genetic risk assessment will be used<br>• Yes – all women will have a genetic risk assessment
Number of women who **need a risk assessment** to **find one** who would develop cancer	Number of women who need a risk assessment to find one woman who would develop cancer in 10 years without intervention	• 25<br>• 160<br>• 300<br>• 425
Number of women who would develop cancer who will be **found as above-average risk through the risk assessment**	Out of every 100 women who would develop cancer in 10 years without intervention, number who will be found as above-average risk through the risk assessment	• 1 out of every 100 women who would develop cancer<br>• 3 out of every 100 women who would develop cancer<br>• 7 out of every 100 women who would develop cancer<br>• 11 out of every 100 women who would develop cancer

Explanation of important words and ideas (1)

Risk assessment

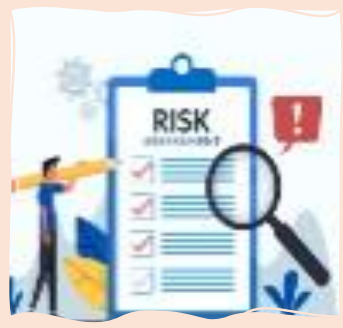

A risk assessment is used to calculate how likely someone is to develop breast cancer in the future. This does not mean that you have, or you will certainly develop breast cancer.

Risk categorisation

Average or population risk: The likelihood that an individual in the general population will develop a specific type of cancer over a defined period of time (often over a lifetime), assuming no special or known risk factors are present.

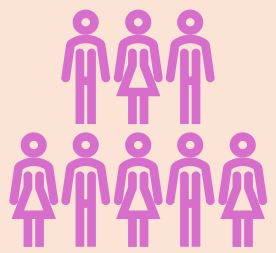

Above-average risk: Refers to a higher-than-average chance of developing cancer, typically due to specific risk factors such as genetic mutations, a strong family history of cancer, certain medical conditions, or lifestyle factors.

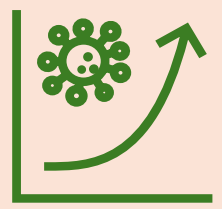

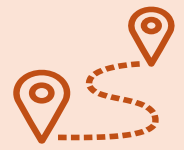

Current care for women under 50

1. Self-present to GP with concerns about risk of breast cancer.

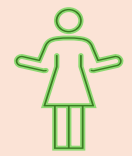

2. GP may refer to specialist services.

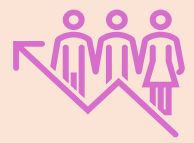

3. Women provide information to clinicians about family history of breast cancer. If necessary, they might be offered genetic test or mammograms.

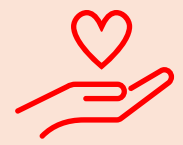

4. Depending on the findings of the risk assessment, women might be offered treatment to lower their risk such as risk reducing medication or risk reducing surgery.

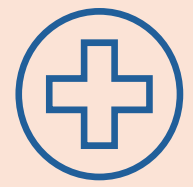

Explanation of important words and ideas (2)

Family history

Giving a family history usually involves considering:

First-degree relatives: parents and children, brothers and sisters, and

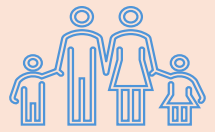

Second-degree relatives: grandparents and grandchildren, aunts/uncles and nieces/nephews.

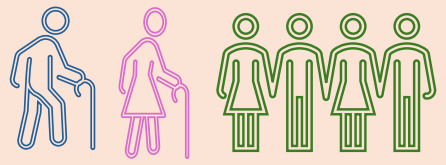

Genetic risk

To do a genetic test, you would need to provide a saliva sample or cheek swab.

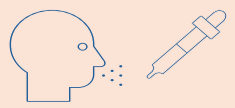

This would allow clinicians to estimate your risk of breast cancer based on your genes.

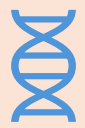

Specifically, this is called on “polygenic risk”, and it considers tiny variations in lots of genes, some of which increase risk and others that protect against breast cancer.

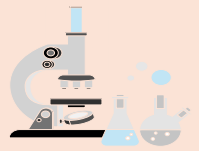

Details of affected family members: completing the questionnaire

Some people might want to contact their relatives for information to help them complete family history questionnaire accurately.

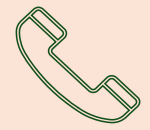

The questionnaire might involve:

a) whether or not anyone has had breast and/or ovarian cancer

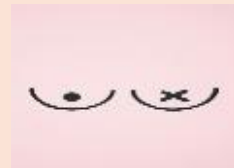

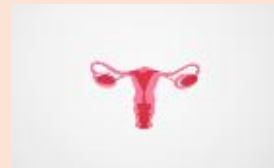

or

b) a detailed family history of both affected and unaffected relatives (all the relatives you have, their age/age when they died, whether each has had breast or ovarian cancer, and when they were diagnosed).

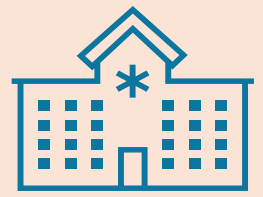

Explanation of important words and ideas (3)

Lifestyle risk factors

Height and weight

Number of children you have had, their sex and at what age

Age at when you started your periods and, if relevant, when you went through the menopause

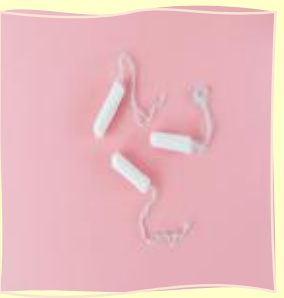

Use of oral contraceptives and hormone replacement therapy (HRT)

Alcohol intake

Ethnicity and whether you have Ashkenazi Jewish ancestry

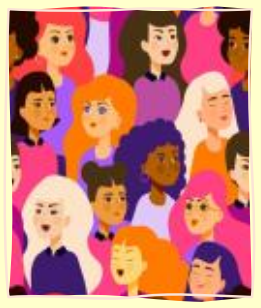

Whether you or other family members have had other rare cancers
