# Supplementary material for: Priorities for breast cancer risk assessment in UK women under age 50: a survey and discrete choice experiment
Source: Br J Cancer. 2026 Aug 4;135(9):1430–8. doi: 10.1038/s41416-026-03546-3 (PMC13675216; doi:10.1038/s41416-026-03546-3)
Supplement: Supplementary file 2 [file 41416_2026_3546_MOESM2_ESM.pdf]

Priorities for breast cancer risk assessment in UK women under age 50: A survey and discrete choice experiment

Table S1. Conditional logistic regression model selection.

	Model description	LL	DF	AIC	BIC	BIC (n=893)	Notes
**Basic model**							
1	Basic clogit model (continuous coding of attributes)	-7075.8	8	14167.6	14232.3	14205.9	Reference BIC
**Adding constant for preference for option 1 or 2 (to consider heuristics preferences)**							
2	Model 1 plus constant for option 1/2	-7073.6	9	14165.2	14238.0	14208.3	No improvement in BIC
**Effects coding each attribute in turn (to check for non-linearity)**							
3a	Model 1 with *N needing risk assessment to find one case* effects coded	-7075.1	10	14170.3	14251.2	14218.2	No improvement in BIC
3b	Model 1 with *N correctly found as above-average risk* effects coded	-7065.4	10	14150.9	14231.8	14198.8	Small improvement in BIC
**Transform each attribute with evidence of non-linearity**							
4a	Model 1 with *N correctly found as above-average risk* transformed - quadratic	-7095.3	8	14206.6	14271.3	14244.9	No improvement in BIC from Model 1
4b	Model 1 with *N correctly found as above-average risk* transformed - log	-7099.8	8	14215.7	14280.4	14254.0	No improvement in BIC from Model 1
4c	Model 1 with *N correctly found as above-average risk* transformed - piecewise (3)	-7074.6	9	14167.3	14240.1	14210.4	No improvement in BIC from Model 1
4d	Model 1 with *N correctly found as above-average risk* transformed - piecewise (7)	-7072.0	9	14162.0	14234.8	14205.1	No *substantial* improvement in BIC from Model 1

AIC: Akaike information criteria; BIC: Bayesian information criteria (adjusted for sample size); DF: Degrees of freedom; NA: not applicable; LL: Log likelihood.

Table S2. Full conditional logistic regression by binary characteristics (subgroup analysis).

a. Age

	<40 years				≥40 years			
	**Coefficient**	**Lower CI**	**Upper CI**	**P value**	**Coefficient**	**Lower CI**	**Upper CI**	**P value**
Current care (ASC)	-0.63	-0.76	-0.50	<0.001	-0.67	-0.81	-0.52	<0.001
Risk assessment initiation								
Opportunistic	-0.56	-0.49	-0.63	<0.001	-0.63	-0.55	-0.71	<0.001
Proactive	0.56	0.49	0.63	<0.001	0.63	0.55	0.71	<0.001
Detailed risk assessment								
All	0.33	0.24	0.43	<0.001	0.23	0.13	0.33	<0.001
Any FH	0.15	-0.02	0.32	0.089	0.21	0.02	0.39	0.026
FH in >1 relative or <40 yrs	-0.48	-0.58	-0.38	<0.001	-0.43	-0.54	-0.33	<0.001
Family history information								
Affected FH	0.12	0.19	0.05	0.001	0.16	0.24	0.08	<0.001
Full FH	-0.12	-0.19	-0.05	0.001	-0.16	-0.24	-0.08	<0.001
Genetic risk assessment								
No	-0.31	-0.24	-0.38	<0.001	-0.39	-0.31	-0.46	<0.001
Yes	0.31	0.24	0.38	<0.001	0.39	0.31	0.46	<0.001
N needing risk assessment to find one case								
	-0.0003	-0.0006	0.0000	0.025	-0.0007	-0.0010	-0.0004	<0.001
N correctly found as above-average risk								
	0.09	0.08	0.10	<0.001	0.09	0.08	0.11	<0.001
	n=474 participants<br>n=12,798 observations<br>Pseudo R^2=0.1922<br>Log likelihood=-3785.9				n=419 participants<br>n=11,313 observations<br>Pseudo R^2=0.2076<br>Log likelihood=-3282.6948			

b. Ethnicity

	Other ethnic groups				White			
	**Coefficient**	**Lower CI**	**Upper CI**	**P value**	**Coefficient**	**Lower CI**	**Upper CI**	**P value**
Current care (ASC)	-0.56	-0.78	-0.34	<0.001	-0.66	-0.77	-0.55	<0.001
Risk assessment initiation								
Opportunistic	-0.55	-0.43	-0.67	<0.001	-0.60	-0.54	-0.66	<0.001
Proactive	0.55	0.43	0.67	<0.001	0.60	0.54	0.66	<0.001
Detailed risk assessment								
All	0.23	0.07	0.38	0.004	0.30	0.23	0.38	<0.001
Any FH	0.01	-0.27	0.29	0.939	0.21	0.07	0.35	0.003
FH in >1 relative or <40 yrs	-0.24	-0.41	-0.07	0.005	-0.51	-0.60	-0.43	<0.001
Family history information								
Affected FH	0.08	0.19	-0.04	0.192	0.15	0.21	0.10	<0.001
Full FH	-0.08	-0.19	0.04	0.192	-0.15	-0.21	-0.10	<0.001
Genetic risk assessment								
No	-0.26	-0.14	-0.37	<0.001	-0.37	-0.31	-0.43	<0.001
Yes	0.26	0.14	0.37	<0.001	0.37	0.31	0.43	<0.001
N needing risk assessment to find one case								
	-0.0003	-0.0008	0.0001	0.187	-0.0005	-0.0007	-0.0003	<0.001
N correctly found as above-average risk								
	0.10	0.09	0.12	<0.001	0.09	0.08	0.10	<0.001
	n=173 participants<br>n=4,671 observations<br>Pseudo R^2=0.1823<br>Log likelihood=-1398.7				n=720 participants<br>n=19,440 observations<br>Pseudo R^2=0.2042<br>Log likelihood=-5665.5			

c. Education

	No degree				Degree			
	Coefficient	Lower CI	Upper CI	P value	Coefficient	Lower CI	Upper CI	P value
Current care (ASC)	-0.66	-0.81	-0.50	<0.001	-0.64	-0.76	-0.52	<0.001
Risk assessment initiation								
Opportunistic	-0.59	-0.50	-0.68	<0.001	-0.59	-0.53	-0.66	<0.001
Proactive	0.59	0.50	0.68	<0.001	0.59	0.53	0.66	<0.001
Detailed risk assessment								
All	0.42	0.31	0.53	<0.001	0.21	0.12	0.30	<0.001
Any FH	0.15	-0.05	0.35	0.144	0.19	0.03	0.35	0.019
FH in >1 relative or <40 yrs	-0.57	-0.69	-0.44	<0.001	-0.40	-0.49	-0.30	<0.001
Family history information								
Affected FH	0.12	0.21	0.04	0.005	0.15	0.22	0.09	<0.001
Full FH	-0.12	-0.21	-0.04	0.005	-0.15	-0.22	-0.09	<0.001
Genetic risk assessment								
No	-0.40	-0.31	-0.48	<0.001	-0.31	-0.25	-0.38	<0.001
Yes	0.40	0.31	0.48	<0.001	0.31	0.25	0.38	<0.001
N needing risk assessment to find one case								
	-0.0001	-0.0004	0.0003	0.739	-0.0007	-0.0010	-0.0005	<0.001
N correctly found as above-average risk								
	0.06	0.05	0.08	<0.001	0.11	0.10	0.12	<0.001
	n=329 participants<br>n=8,883observations<br>Pseudo R^2=0.2015<br>Log likelihood=-2597.5				n=564 participants<br>n=15,228 observations<br>Pseudo R^2=0.2030<br>Log likelihood=-4444.3			

d. Self-perceived socioeconomic status

	Higher				Lower			
	Coefficient	Lower CI	Upper CI	P value	Coefficient	Lower CI	Upper CI	P value
Current care (ASC)	-0.64	-0.75	-0.53	<0.001	-0.68	-0.90	-0.46	<0.001
Risk assessment initiation								
Opportunistic	-0.60	-0.54	-0.66	<0.001	-0.55	-0.43	-0.68	<0.001
Proactive	0.60	0.54	0.66	<0.001	0.55	0.43	0.68	<0.001
Detailed risk assessment								
All	0.28	0.20	0.35	<0.001	0.32	0.16	0.48	<0.001
Any FH	0.17	0.03	0.30	0.019	0.20	-0.08	0.49	0.165
FH in >1 relative or <40 yrs	-0.44	-0.53	-0.36	<0.001	-0.52	-0.70	-0.35	<0.001
Family history information								
Affected FH	0.13	0.19	0.07	<0.001	0.19	0.32	0.07	0.002
Full FH	-0.13	-0.19	-0.07	<0.001	-0.19	-0.32	-0.07	0.002
Genetic risk assessment								
No	-0.34	-0.28	-0.40	<0.001	-0.35	-0.23	-0.48	<0.001
Yes	0.34	0.28	0.40	<0.001	0.35	0.23	0.48	<0.001
N needing risk assessment to find one case								
	-0.0004	-0.0006	-0.0002	<0.001	-0.0006	-0.0011	-0.0002	0.005
N correctly found as above-average risk								
	0.09	0.08	0.10	<0.001	0.10	0.08	0.12	<0.001
	n=723 participants<br>n=19,521 observations<br>Pseudo R^2=0.1968<br>Log likelihood=-5741.6				n=170 participants<br>n=4,590 observations<br>Pseudo R^2=0.2077<br>Log likelihood=-1331.8			

e. Family history of cancer

	No family history				Family history			
	Coefficient	Lower CI	Upper CI	P value	Coefficient	Lower CI	Upper CI	P value
Current care (ASC)	-0.60	-0.71	-0.48	<0.001	-0.73	-0.90	-0.55	<0.001
Risk assessment initiation								
Opportunistic	-0.60	-0.54	-0.67	<0.001	-0.56	-0.47	-0.66	<0.001
Proactive	0.60	0.54	0.67	<0.001	0.56	0.47	0.66	<0.001
Detailed risk assessment								
All	0.32	0.23	0.40	<0.001	0.22	0.10	0.34	0.001
Any FH	0.13	-0.02	0.28	0.098	0.28	0.06	0.50	0.013
FH in >1 relative or <40 yrs	-0.44	-0.54	-0.35	<0.001	-0.50	-0.64	-0.37	<0.001
Family history information								
Affected FH	0.14	0.21	0.08	<0.001	0.13	0.23	0.04	0.005
Full FH	-0.14	-0.21	-0.08	<0.001	-0.13	-0.23	-0.04	0.005
Genetic risk assessment								
No	-0.36	-0.30	-0.43	<0.001	-0.32	-0.23	-0.42	<0.001
Yes	0.36	0.30	0.43	<0.001	0.32	0.23	0.42	<0.001
N needing risk assessment to find one case								
	-0.0005	-0.0007	-0.0002	<0.001	-0.0005	-0.0009	-0.0002	0.005
N correctly found as above-average risk								
	0.08	0.07	0.09	<0.001	0.11	0.09	0.12	<0.001
	n=596 participants<br>n=16,092 observations<br>Pseudo R^2=0.1930<br>Log likelihood=-4755.8				n=277 participants<br>n=7,479 observations<br>Pseudo R^2=0.2090<br>Log likelihood=-2166.4			

ASC: alternative specific constant; CI: confidence interval; FH: family history (of breast or ovarian cancer); N: number of women.

Note: direct comparisons between subgroups should be interpreted with caution due to potential scale heterogeneity.

Table S3. Participants' ease of completing the DCE and perceived impact of different attributes on decision-making.

	N	(%)
**Total**	**893**	**100**
**Ease of completing the DCE**		
Difficult	539	60.4
Easy	354	39.6
**Number of attributes considered**		
1 or 2	445	49.8
3 or 4	371	41.5
5 or 6	77	8.6
**Ranking of attributes**		

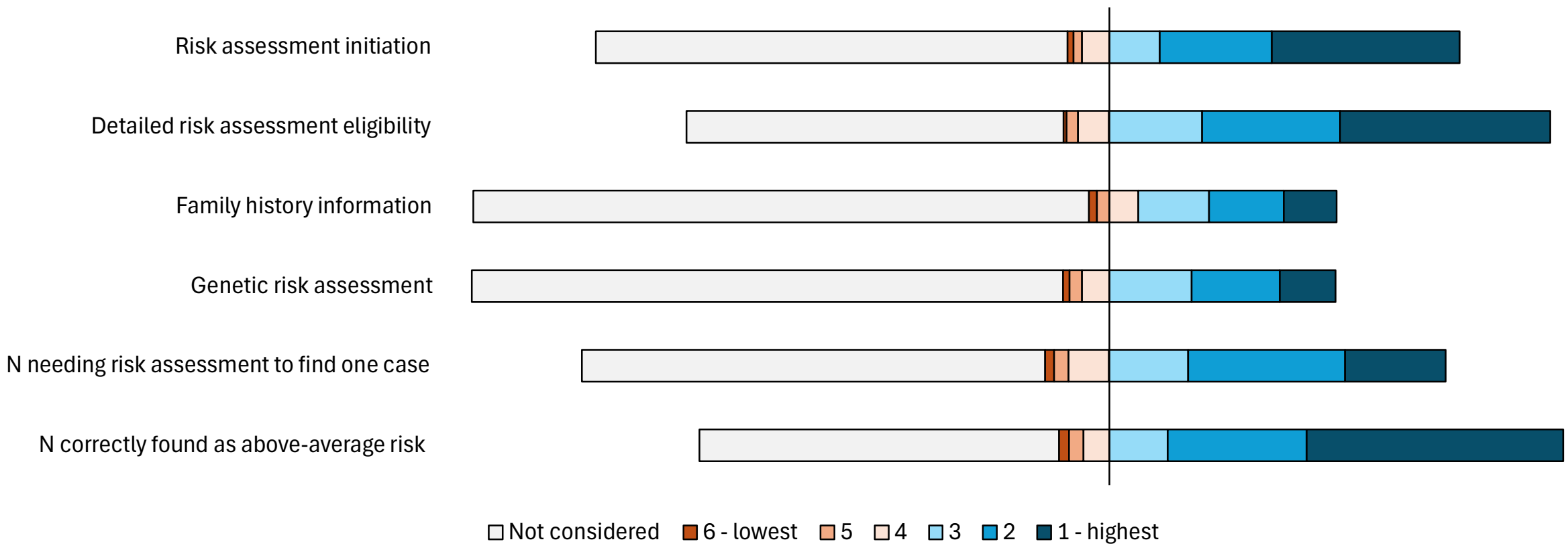

		Risk assess-ment initiation	Detailed risk assess-ment eligibility	Family history information	Genetic risk assess-ment	N needing risk assess-ment to find one case	N correctly found as above-average risk
		**N (%)**	**N (%)**	**N (%)**	**N (%)**	**N (%)**	**N (%)**
1 - highest	■	194 (21.7)	217 (24.3)	55 (6.2)	58 (6.5)	104 (11.6)	265 (29.7)
2	■	116 (13.0)	143 (16.0)	77 (8.6)	91 (10.2)	162 (18.1)	144 (16.1)
3	■	52 (5.8)	96 (10.8)	73 (8.2)	85 (9.5)	82 (9.2)	60 (6.7)
4	■	28 (3.1)	32 (3.6)	30 (3.4)	28 (3.1)	42 (4.7)	27 (3.0)
5	■	9 (1.0)	12 (1.3)	13 (1.5)	13 (1.5)	15 (1.7)	15 (1.7)
6 - lowest	■	6 (0.7)	3 (0.3)	8 (0.9)	7 (0.8)	9 (1.0)	10 (1.1)
Not considered	□	488 (54.6)	390 (43.7)	637 (71.3)	611 (68.4)	479 (53.6)	372 (41.7)

Figure S1. Participants' comfort with aspects of detailed risk assessments.

How comfortable would you be...

... giving a detailed family history of affected and unaffected relatives

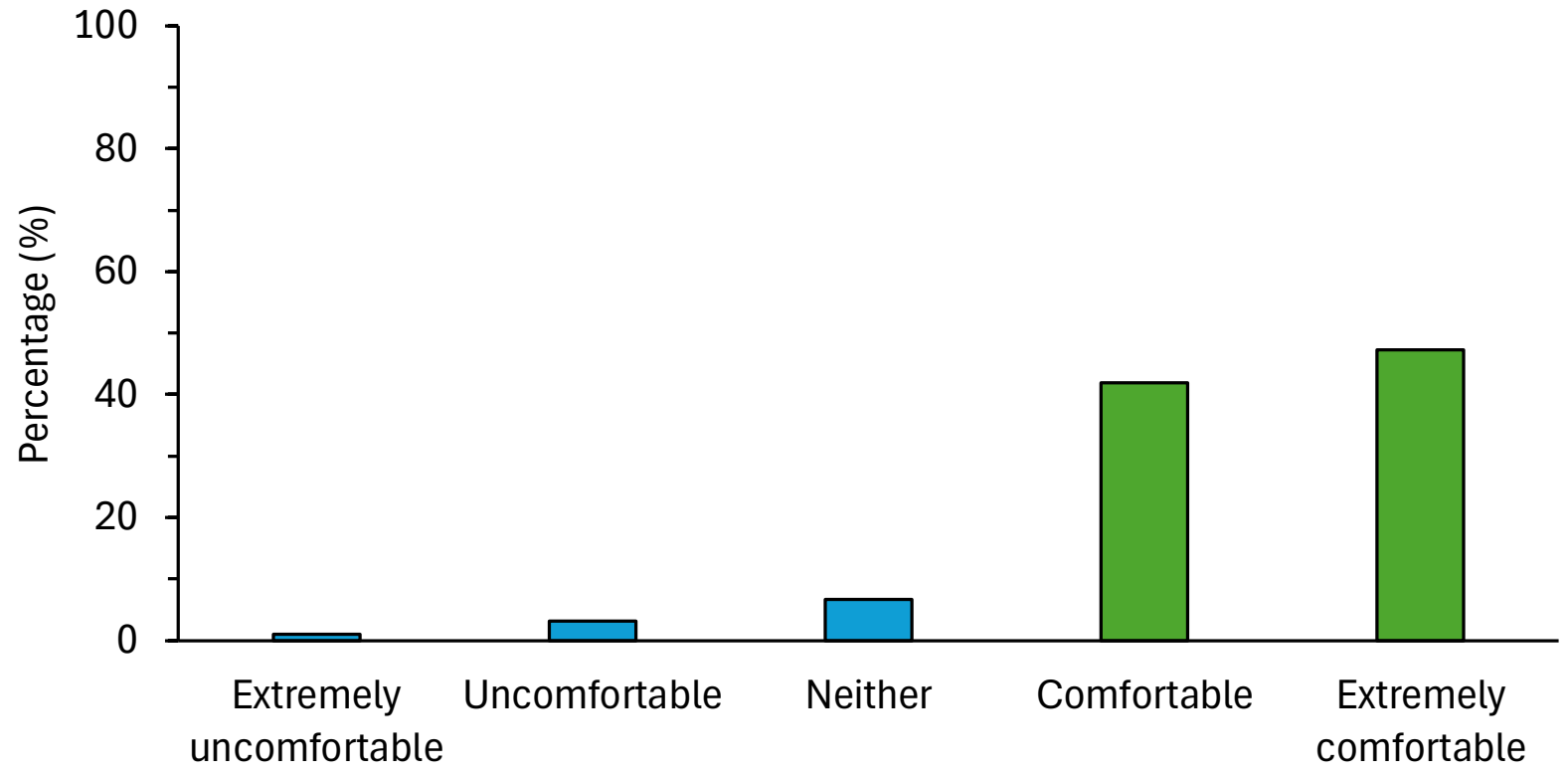

... completing a risk factors questionnaire

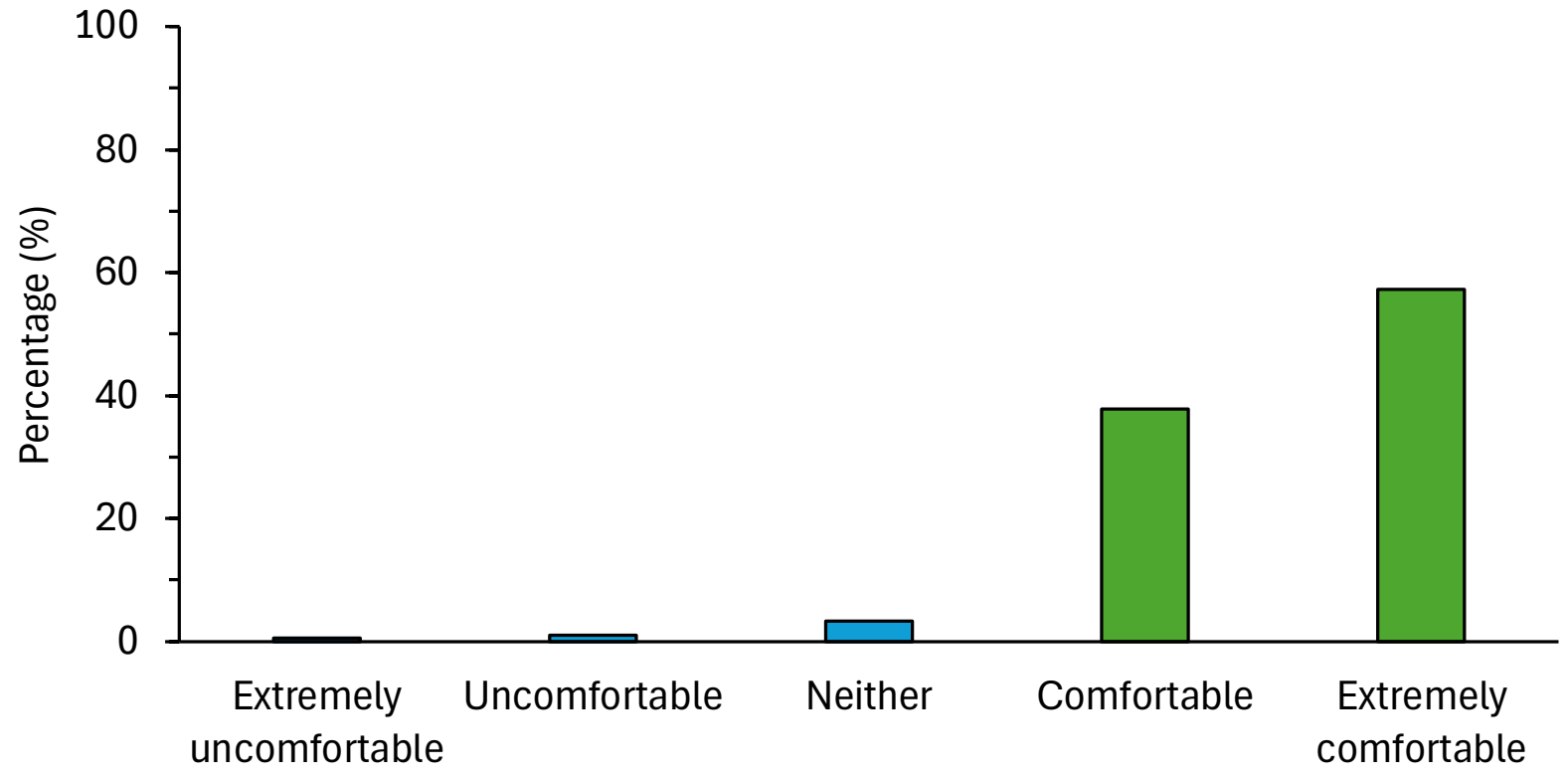

... completing a genetic breast cancer risk assessment

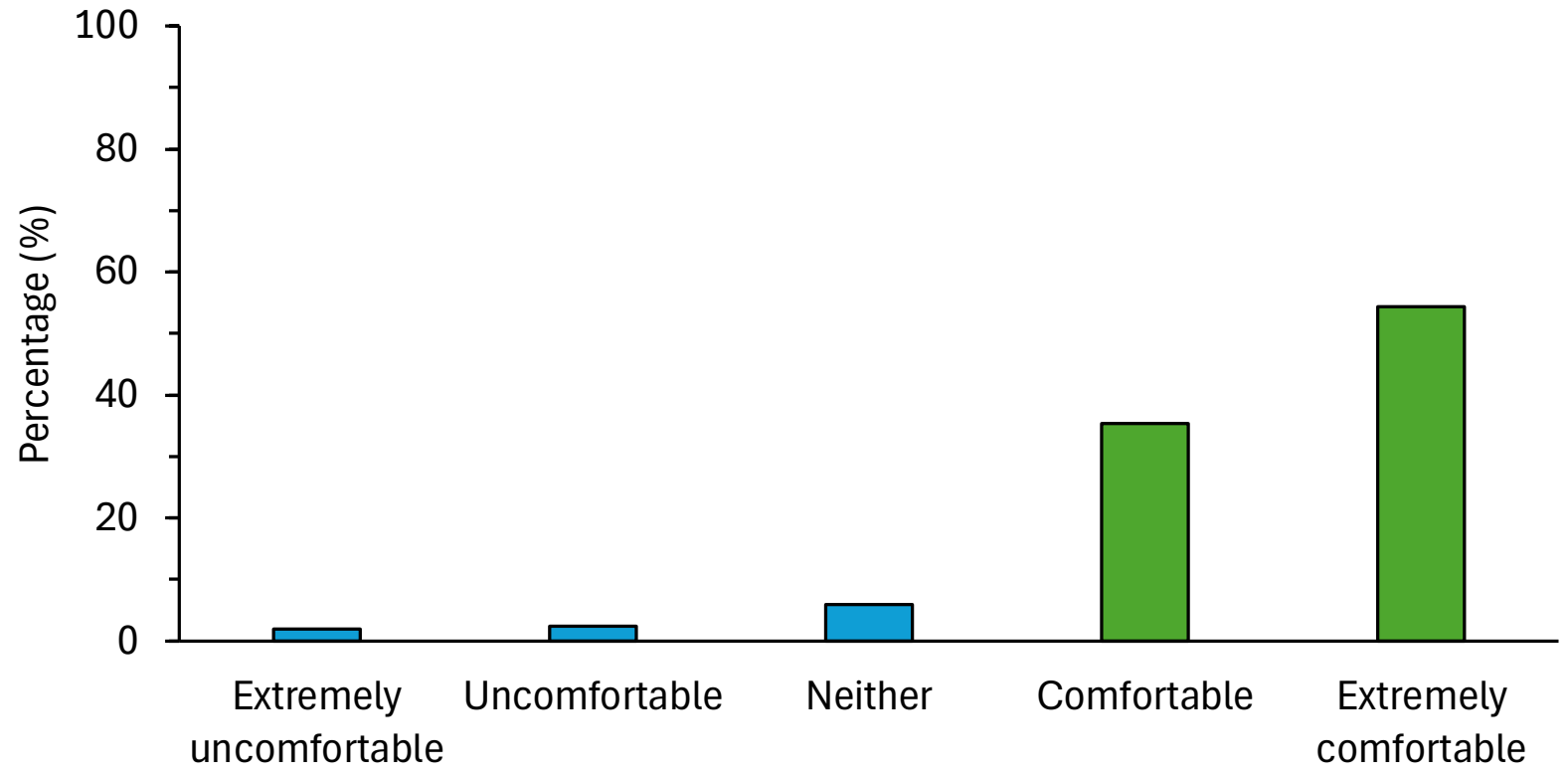

Participants' comfort with each statement (n=893).

Figure S2. Acceptability of breast cancer risk assessments for younger women.

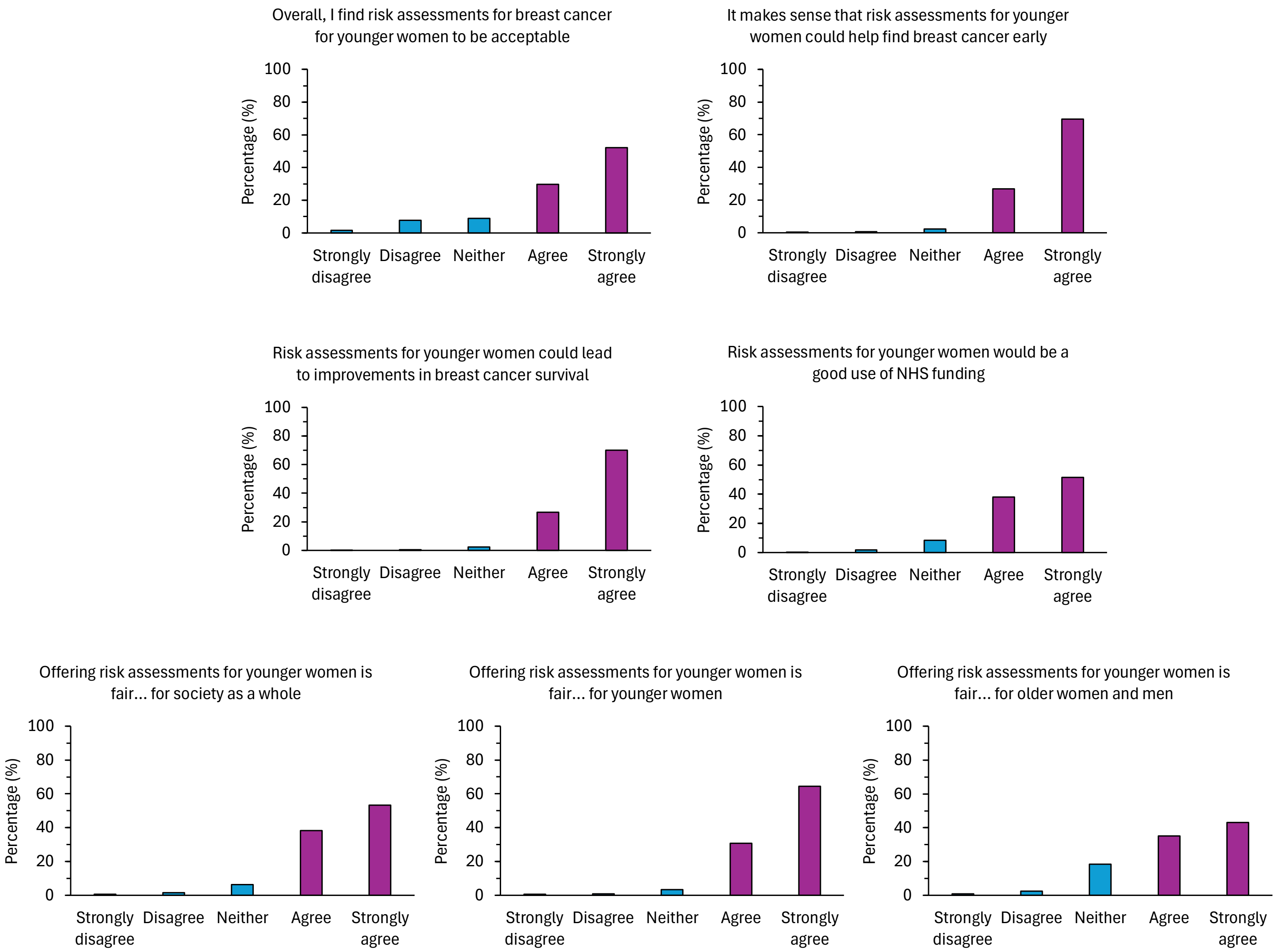

Participants' agreement with each statement (n=893).
