# Supplementary material for: Priorities for breast cancer risk assessment in UK women under age 50: a survey and discrete choice experiment
Source: Br J Cancer. 2026 Aug 4;135(9):1430–8. doi: 10.1038/s41416-026-03546-3 (PMC13675216; doi:10.1038/s41416-026-03546-3)
Supplement: Supplementary file 3 [file 41416_2026_3546_MOESM3_ESM.docx]

DIscrete choice experiment REporting ChecklisT (DIRECT checklist)

Priorities for breast cancer risk assessment in UK women under age 50: A discrete choice experiment (DCE)

| Section Item | | Page and paragraph |
| --- | --- | --- |
| Purpose and rationale | |  |
| 1 | Describe the real-world context and decision-maker that the hypothetical choice context seeks to replicate or inform | Page 3 4^th^ paragraph |
| 2 | Provide a rationale for using a DCE to answer the research question | Page 4 2^nd^ paragraph |
| Attributes and levels^a^ | |  |
| 3 | Describe how attributes and levels were derived (e.g. literature review, interviews, focus groups, expert input) | Page 5 DCE design |
| 4 | Provide the final list of attributes and levels | Table 1 |
| Experimental design | |  |
| 5 | Report the number of alternatives per choice set and whether they were labelled or unlabelled | Page 6 2^nd^ paragraph |
| 6 | Describe response options (e.g. forced choice, opt-out, status quo) | Page 6 3^rd^ paragraph |
| 7 | Describe the type of experimental design (e.g. orthogonal, D-efficient, Bayesian efficient, partial profile) | Page 6 2^nd^ paragraph |
| 8 | Describe which effects are identified in the design (e.g. main effects, higher order interactions, functional form) | Page 7 Analysis |
| 9 | Describe the number of choice sets, blocks and choice sets per block | Page 6 2^nd^ paragraph |
| 10 | Indicate how the experimental design was obtained (software, catalogue, other) | Page 6 2^nd^ paragraph |
| Survey design | |  |
| 11 | Provide a sample choice set and the instructions and background information given to respondents (e.g. providing the survey as an appendix) | Supplementary File 1 |
| 12 | Report any randomisation (e.g. choice set order, attribute order, alternative order, framing effects) | Page 6 2^nd^ paragraph |
| 13 | Describe what was checked in piloting (e.g. understanding, respondent burden, timing, wording) | Page 7 1^st^ paragraph |
| 14 | Report whether information from the pilot was used to update the experimental design (e.g. priors, functional form of attributes) or survey design | Page 7 1^st^ paragraph |
| Sample and data collection | |  |
| 15 | Report respondent inclusion/exclusion criteria | Page 4 Participants and recruitment |
| 16 | Describe how data were collected (e.g. mail, personal interview, web survey) | Page 5 Survey design |
| 17 | Report the response rate or cooperation rate, if possible | Page 8 Responses and participant characteristics |
| 18 | Report the final sample size and how the sample size was determined | Page 8 Responses and participant characteristics and Page 5 2^nd^ paragraph |
| 19 | Describe respondent characteristics and representativeness of target population, if known | Participant characteristics |
| Econometric analysis | |  |
| 20 | Indicate coding of data (e.g. effects, dummy, continuous) including definitions | Page 7 Analysis |
| 21 | Report whether any respondents were removed and why (e.g. suspected fraudulent responses, rationality tests) | Page 7 Analysis |
| 22 | Provide the rationale for model choice (e.g. conditional logit, mixed logit, latent class) and assumptions (e.g. error variance) | Page 7 Analysis |
| 23 | Report model specification | Page 7 Analysis and Page 8 Model selection |
| Reporting of results | |  |
| 24 | Report the model performance, goodness of fit (if comparing models) | Table S1 |
| 25 | Describe methods used for analysis of model results (e.g. calculation of marginal rate of substitution, attribute relative importance, welfare gain) | Page 7 Analysis |
| 26 | Report measures of precision for the output(s) of interest (e.g. confidence intervals) and how these were derived | Page 7 Analysis and throughout results |
